# Increasing trend of radiographic features of knee osteoarthritis in rheumatoid arthritis patients before total knee arthroplasty

**DOI:** 10.1038/s41598-022-14440-2

**Published:** 2022-06-21

**Authors:** Ryutaro Takeda, Takumi Matsumoto, Yuji Maenohara, Yasunori Omata, Hiroshi Inui, Yuichi Nagase, Takuji Nishikawa, Sakae Tanaka

**Affiliations:** 1 Department of Orthopaedic Surgery, Faculty of Medicine, The University of Tokyo, 7-3-1 Hongo, Bunkyo-ku, Tokyo, 113-8655 Japan; 2 Department of Rheumatic Surgery, Tokyo Metropolitan Tama Medical Center, 2-8-29 Musashidai, Fuchu-city, Tokyo, 183-8524 Japan; 3 Department of Rheumatology, Tokyo Metropolitan Bokutoh Hospital, 4-23-15 Koutoubashi, Sumida-ku, Tokyo, Japan

**Keywords:** Medical research, Rheumatology

## Abstract

To investigate the trend and factors related to the occurrence of osteoarthritis (OA)-like features on knee radiographs of rheumatoid arthritis (RA) patients undergoing total knee arthroplasty (TKA) in the recent decades. To classify antero-posterior knee radiographs into ‘RA’ and ‘OA-like RA’ groups, a deep learning model was developed by training the network using knee radiographs of end-stage arthropathy in RA patients obtained during 2002–2005 and in primary OA patients obtained during 2007–2009. We used this model to categorize 796 knee radiographs, which were recorded in RA patients before TKA during 2006–2020, into ‘OA-like RA’ and ‘RA’ groups. The annual ratio of ‘OA-like RA’ was investigated. Moreover, univariate and multivariate analyses were performed to identify the factors associated with the classification as OA-like RA using clinical data from 240 patients. The percentage of ‘OA-like RA’ had significant increasing trend from 20.9% in 2006 to 67.7% in 2020. Higher body mass index, use of biologics, and lower level of C-reactive protein were identified as independent factors for ‘OA-like RA’. An increasing trend of knee radiographs with OA-like features was observed in RA patients in the recent decades, which might be attributed to recent advances in pharmacotherapy.

## Introduction

Rheumatoid arthritis (RA) is a chronic inflammatory disease characterized by multiple joint destruction due to persistent synovitis, which subsequently causes functional disability. Pharmacological therapy is the mainstay of treatment to control inflammation for joint preservation in RA patients^1^. In the past 20 years, significant advancement in pharmacological therapy represented by the developments of biological or targeted synthetic disease modifying anti-rheumatic drugs (bDMARDs or tsDMARDs) and treat-to-target strategy aiming at tight control of disease activity has dramatically ameliorated the management of RA^2^. A recent cohort study reported that more than half of the RA patients achieved a remission^3^.

According to some nation-wide databases, the incidence of joint replacement surgery in RA patients has declined with the advances in pharmacological therapy^4–6^. On the other hand, the number of total knee arthroplasty (TKA) among the general population has been increasing in the recent two decades, which suggests an increasing demand of TKA due to aging population and subsequent increase in the incidence of knee osteoarthritis (OA)^7–9^. Therefore, the population and characteristics of RA patients who require TKA are also expected to change.

A few previous studies investigating radiographs of RA patients have reported a correlation between low levels of C-reactive protein (CRP) and increased formation of periarticular osteophytes^10–12^. Although these studies suggested that RA patients with well-controlled disease activity tended to show the radiographic characteristics of OA, they focused only on osteophytes, ignoring other characteristics such as subchondral bone sclerosis, cysts, joint space narrowing. Due to an increase in the percentage of patients with well-controlled disease activity, the number of RA patients showing the characteristics of OA (OA-like RA) is expected to increase.

In the current study, we developed a convolutional neural network (CNN) model to objectively judge knee radiographs for classification as either OA or RA. This is because analysis between conventional RA and OA-like RA is highly subjective and is dependent on the person that classifies them. The CNN model was developed using deep learning and was trained using knee radiographs of conventional RA patients (recorded before 2005) and primary OA patients (not secondary to RA).

The primary aim of the current study was to investigate the annual trend of radiographic features of OA in RA patients who underwent TKA from 2006 to 2020 using our original developed CNN model. The second aim was to identify the factors associated with the occurrence of radiographic features of OA in RA patients.

## Methods

### Development of the deep learning model

**Figure 1:**
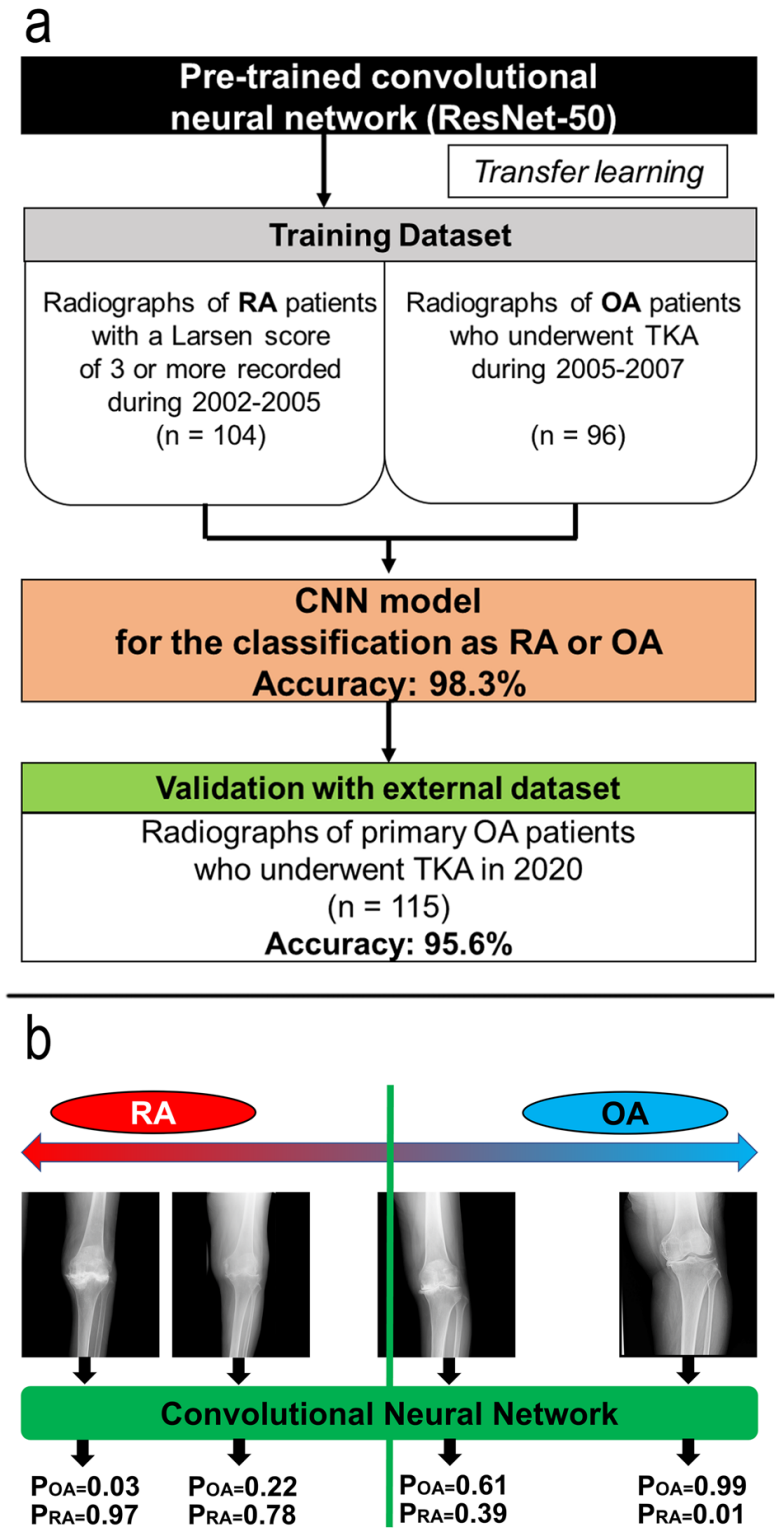
(**a**) Development of the deep learning model. An overview of the training and validation of the model. RA, rheumatoid arthritis; OA, osteoarthritis; TKA, total knee arthroplasty. (**b**) Concept of classification by convolutional neural network. The convolutional neural network calculates the possibility of osteoarthritis and rheumatoid arthritis based on the input radiographs. If the probability of osteoarthritis was higher than 0.5, the radiograph was classified into ‘OA’. POA, probability of osteoarthritis; PRA, probability of rheumatoid arthritis.

An overview of the development of the deep learning model is shown in Fig. 1a. The CNN model developed in the current study is available in GitHub at https://github.com/takedarort/publish/releases/tag/v1.0. Note that the CNN model was not used for the diagnosis of RA because the model was only developed for the current study using the radiographs of patients with end-stage knee arthritis requiring TKA at our institutions. The generalization performance of the CNN model for other purposes is not guaranteed.

#### Model

We built the deep convolutional neural network (CNN) model to classify antero-posterior weightbearing knee radiographs as RA and OA using transfer learning. To use transfer learning in the current study, ResNet-50 (Microsoft Corporation, WA, USA), which is a CNN with 117 layers pre-trained on more than a million images from the ImageNet database, was retained^13^. The fully connected layer and classification layer of the CNN were modified to classify radiographs as RA or OA based on a comparison of their probabilities (Fig. 1b). After reconstruction, the CNN was trained using knee radiographs. Since the input size of the CNN was 224*224, radiographs were resized to 224*224 pixels before using them for training and validation. Adaptive moment estimation (Adam) was used as a method to optimize the loss function of the CNN^14^. The learning rate, which is the step size at each iteration of tuning parameter to minimize the loss function, was biased by ten times for the replaced layer to protect the feature extraction potential of the network; the learning rate for the transferred layers and replaced layer was set to 0.0001 and 0.001, respectively. The batch size was set to 8. The number of epochs was fixed at 13. These hyperparameters were tuned by one of the authors to maximize the accuracy of the CNN model.

Deep learning toolbox in MATLAB 2020a (MathWorks, CA, USA) was used in image processing and the development of the CNN model.

#### Dataset

We retrospectively reviewed all RA patients who were admitted to the department of orthopedic surgery of the University of Tokyo Hospital from 2002 to 2005. All the RA patients included in the training dataset met the diagnostic criteria proposed by American College of Rheumatology in 1987^15^. Total 104 antero-posterior weightbearing knee radiographs with a Larsen score of 3 or more from these patients were used as RA for training. The Larsen score of 3 means medium joint destruction with bone erosion which was the characteristics of chronic synovitis. To avoid overlap between the training dataset and test dataset, the data from patients who underwent TKA from 2006 to 2020 were excluded from the training dataset. There are two reasons for designating 2002–2005 as our data collection period: For one thing, we had planned to collect at least 100 radiographs. Another is, the radiographs before 2002 were not digitally stored in the institution. Although it was 2003 when the first bDMARDs, infliximab, was approved for treatment of RA in Japan, there was no patient in the training dataset with a history of usage of bDMARDs. Therefore, the training dataset was considered to represent the radiographs of pre-bDMARDs period. Total 96 antero-posterior knee radiographs of all consecutive patients who underwent TKA due to primary OA at the same hospital from 2007 to 2009 were used as OA for training. There were two reasons for choosing this period: For one thing, the database of TKA for OA patients has been saved as electronic file since 2007. Another is, triennium was enough to obtain the equivalent number of OA-labeled data to the RA dataset. In all radiographs of the training dataset, regions containing letter and the white area resulted from the aperture of irradiation were manually covered with black rectangles to train the CNN correctly. To avoid arbitrary modifications to the radiograph, image processing was not performed for the dataset in the validation session or the dataset to investigate the ratio of OA-like RA from 2006 to 2020.

#### Data augmentation

To overcome the problem of limited training data, images were augmented by applying a number of random image transformation: horizontal and vertical translation up to 12 pixels, rotation by an angle up to 12 °, horizontal flipping, and image scaling from 0.7 to 1.42.

The augmented dataset containing 200 radiographs was obtained every time the entire dataset was used for training. Since the number of epoch, which is the number of times the entire dataset was processed for training, was set to 13, a total of 2600 (= 200×13) augmented images were used for training the model.

#### Validation of the model

In the current study, the model was validated once per epoch to monitor the training progress. The dataset of 200 radiographs was randomly split into a training dataset (140 radiographs) and a validation dataset (60 radiographs) before each training epoch. Training accuracy was calculated using the validation dataset after each training epoch. After all training epochs, the model achieved an accuracy of 98.3%, sensitivity of 96.7 %, specificity of 100%, precision of 100% and F1-score of 98.3%, when ‘OA’ was defined as positive.

After the model was developed, further validation using a dataset independent of the training dataset was performed to evaluate the generalization capability. The model might fit recently recorded radiographs, because the training data were recorded during 2002-2009. Therefore, to eliminate the possibility of overfitting to the training dataset, the model was further validated using 115 radiographs obtained from all patients who underwent TKA due to primary OA in 2020 at the University of Tokyo Hospital. As a result, 95.6% of the dataset were correctly classified as OA, suggesting that the model fitted not only to the training dataset but also to the recently obtained radiographs independent of the training dataset.

**Figure 2:**
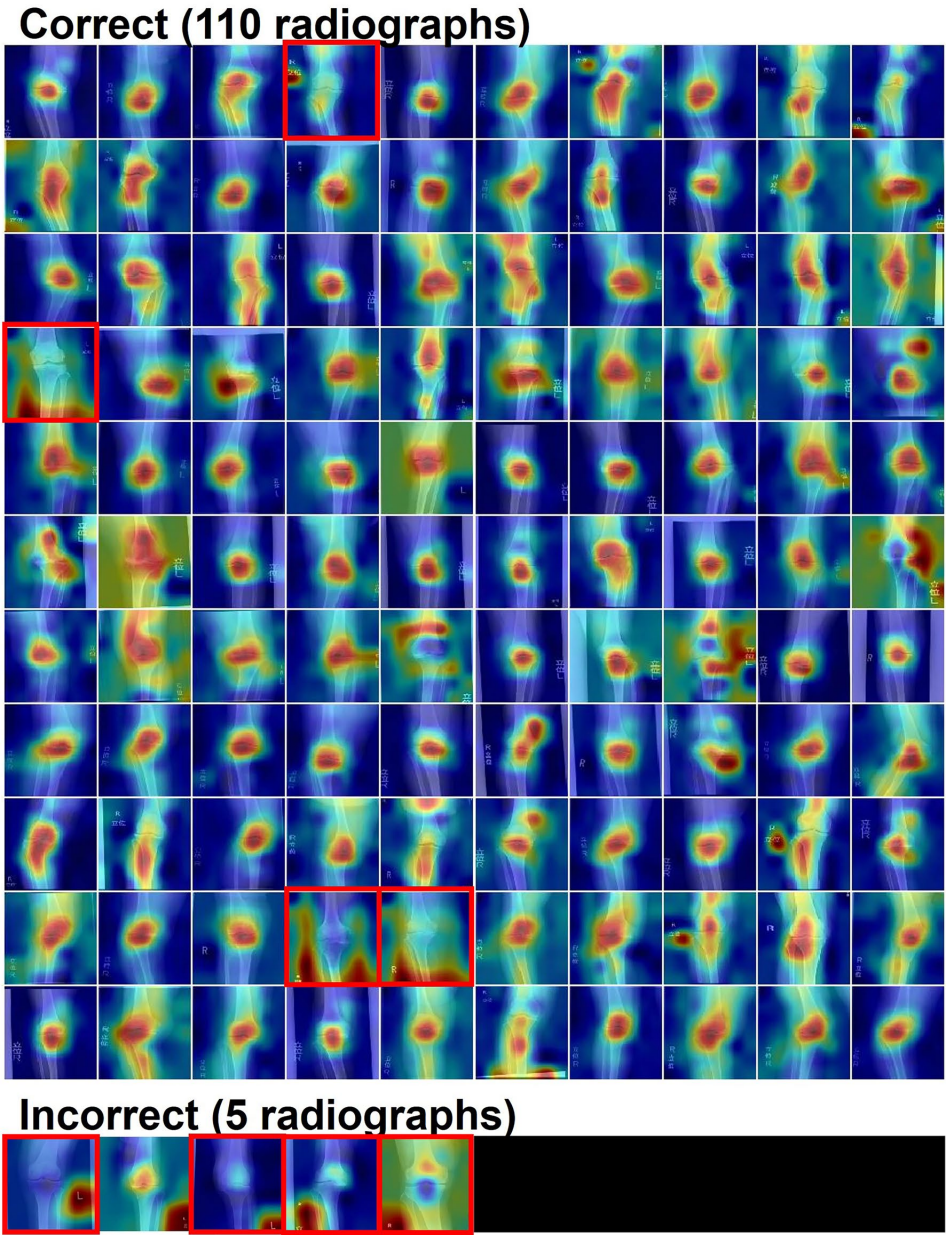
Validation of the model with Grad-CAM on the datasets containing 115 radiographs of primary osteoarthritis. The red-colored region in the heatmap demonstrates the area with high contributed to the classification by the model. In 93.0% (107/115) radiographs, the class-discriminating area included joint areas. Eight radiographs where the class-discriminating area did not include joint area were surrounded by red rectangles. Correct; radiographs which were correctly classified as ‘OA’, Incorrect; radiographs which were incorrectly classified as ‘RA’.

Gradient-weighted Class Activation Mapping (Grad-CAM) was performed, on the previously mentioned 115 radiographs, to confirm whether the CNN model predicted the class, based on the joint area of the radiographs. Grad-CAM is a technique used to generate heatmaps to highlight the area contributing to the classification^16^. After reviewing the heatmaps, the class-discriminating area included joint areas in 93.0% (107/115) of radiographs, suggesting that the model focused on the knee joints (Fig. 2).

### Trend investigation of the ratio of ‘OA-like RA’

**Figure 3:**
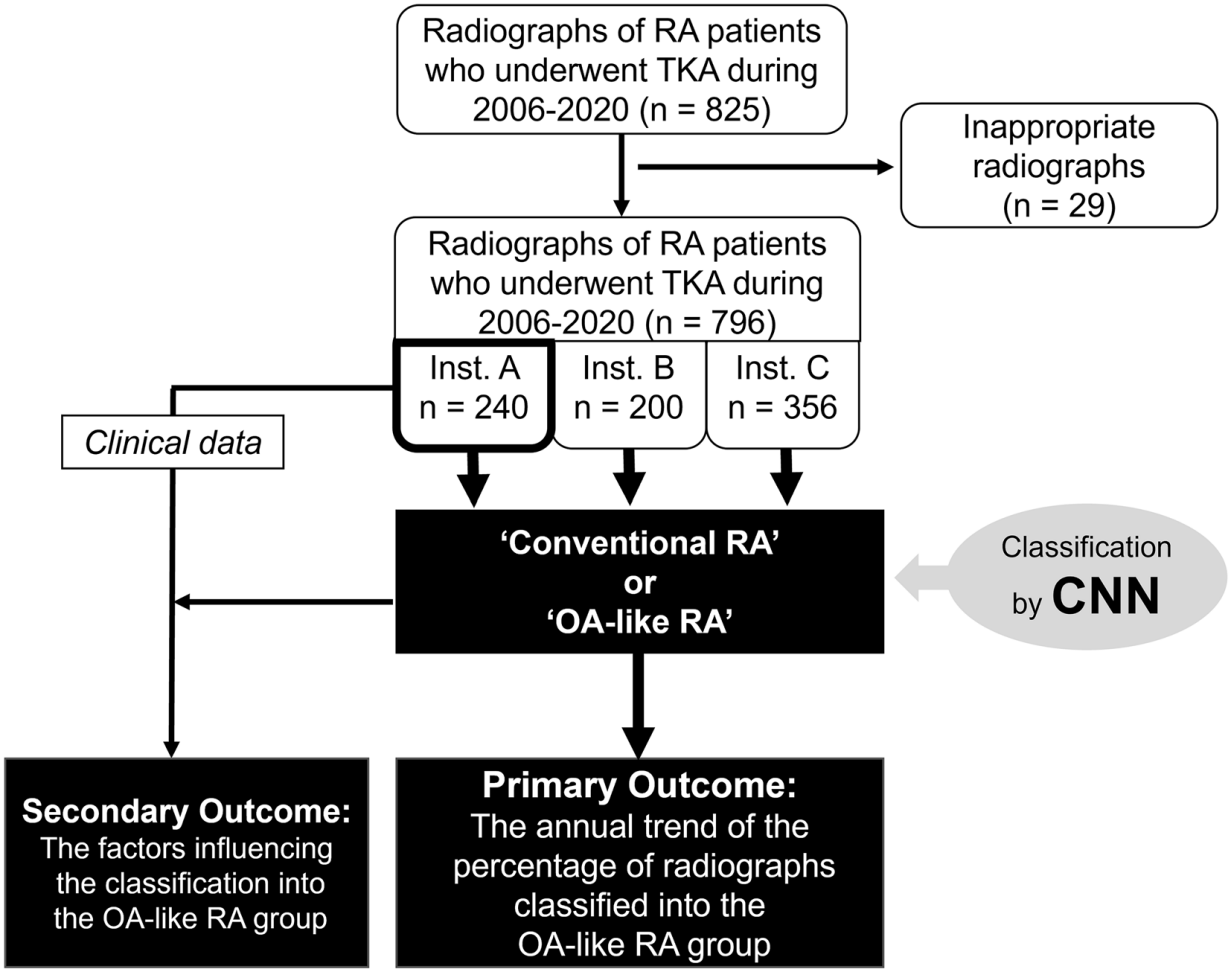
Flowchart of the study. Inst. A, B, and C represent three institutions involved in the current study. RA, rheumatoid arthritis; OA, osteoarthritis; TKA, total knee arthroplasty; CNN, convolutional neural network.

The flowchart of the current study is presented in Fig. 3.

#### Radiographs to be classified into ‘OA-like RA’ or ‘conventional RA’

Surgical databases of three institutions (the University of Tokyo Hospital, Tokyo Metropolitan Tama Medical Center, and Tokyo Metropolitan Bokutoh Hospital) were retrospectively reviewed, and RA patients who underwent TKA from 2006 to 2020 were enrolled in the current study. It was confirmed that all the RA patients included in the current study met the diagnostic criteria for RA proposed by American College of Rheumatology in 1987 or the criteria proposed by American College of Rheumatology and European League Against Rheumatism in 2010^15,17^. Patients who were diagnosed with osteonecrosis of the femoral condyle in addition to RA were excluded. The patients who underwent bilateral TKA were regarded as two separate cases. The standing antero-posterior preoperative knee radiograph of each case was obtained as a DICOM file. In 57 cases, knee radiographs included both the knees; such radiographs were automatically separated along the midline, and one of the knees was covered with black rectangle to regain the original pixel-size. Total 825 radiographs from 653 patients were selected. Among them, 29 radiographs were excluded due to the following reasons: partially containing the contralateral knee, containing implants, and undividable using automatic separation method. Finally, 796 radiographs from 624 patients were included in the analysis. Among them, 88 radiographs were collected from male patients, and 708 radiographs were collected from female patients; the mean age of the study population was 67.6 ± 9.5 years. Radiographs were divided into 15 subgroups according to the year of surgery, i.e., each year from 2006 to 2020.

#### Definition of ‘OA-like RA’ and ‘conventional RA’

Radiographs were classified as RA and OA by the deep CNN model described above. ’OA-like RA’ was defined as the radiograph classified into ‘OA’ by the CNN and ‘conventional RA’ was defined as the radiograph classified into ‘RA’ by the CNN.

#### Primary and secondary outcomes

The primary outcome was the ratio of OA-like RA to the sum of conventional RA and OA-like RA.

The secondary outcome was the clinical factors associated with the classification as OA-like RA. We conducted univariate and multivariate analyses using 240 radiographs from 192 patients who underwent TKA at the University of Tokyo Hospital. For the analyses, the clinical data including, sex, age, body mass index (BMI), duration of disease, dosage of prednisolone (PSL), dosage of methotrexate (MTX), use of bDMARDs, level of C-reactive protein (CRP), erythrocyte sedimentation rate (ESR), presence of anti-cyclic citrullinated peptide antibody (ACPA), and Disease Activity Score-28 for RA with CRP (DAS28-CRP) were extracted via medical chart review. The use of bDMARDs in this article is defined as the continuous use of bDMARDs over the last 6 months before knee radiography was performed. This cutoff value of 6 months was adopted because some previous studies have reported an effect of bDMARDs on the radiographic progression of structural joint damage 6 months after administration^18,19^.

### Statistics

All statistical analyses were conducted using MATLAB 2020a (MathWorks, MA, USA). The level of statistical significance was set at *p* < 0.05. The annual trend of the ratio of OA-like RA to the sum of conventional RA and OA-like RA from 2006 to 2020 was assessed by the Cochran—Armitage trend test. The linear regression model was used to analyze the trends of the number of TKA performed at three institutions from 2006 to 2020. To identify the clinical factors associated with the classification as OA-like RA, univariate analysis was performed using the clinical data described above. The assumption of normality was assessed for all continuous variables by the Kolmogorov—Smirnov test. The t-test and Mann—Whitney U-test was used for normally distributed continuous variables and interval or not normally distributed variables, respectively. The chi-square test was performed for categorical variables.

Based on the univariate analysis, multivariate logistic regression analysis was conducted to predict the classification into the OA-like RA group. To assess the statistical significance of each explanatory variables, the odds ratio and their 95% confidence interval (CI) were calculated.

### Ethics consideration

The current study was conducted in accordance with the Declaration of Helsinki and was approved by the research ethics committee of The University of Tokyo hospital (No. 2674-4), the ethics committee of Tokyo Metropolitan Tama Medical Center (No. 30-52), and the ethics committee of Tokyo Metropolitan Bokutoh Hospital (No. 02-122). Written informed consent was obtained from participants at the University of Tokyo. The boards of Tokyo Metropolitan Tama Medical Center and Metropolitan Bokutoh Hospital waived the requirement for patient’s informed consent because of the anonymous nature of the data.

## Results

### The annual trend of the number of TKA performed and the percentage of OA-like RA

**Figure 4:**
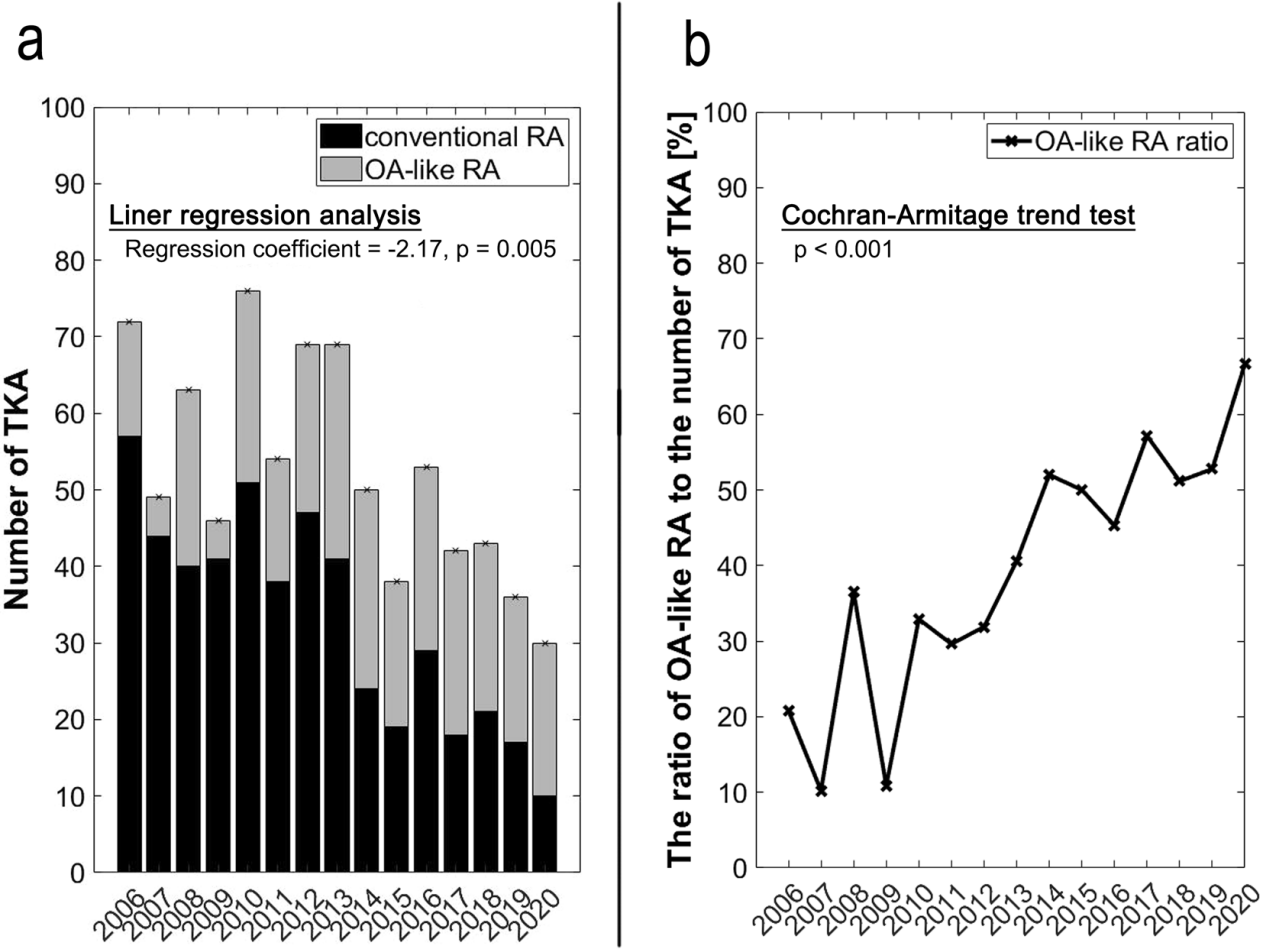
(**a**) The annual trend of the number of total knee arthroplasty (TKA) performed in rheumatoid arthritis (RA) patients. The OA-like RA group included RA patients whose radiographs were classified as osteoarthritis (OA) by the artificial intelligence (AI) model. The conventional RA group consisted of RA patients whose radiographs were classified as RA by the AI. (**b**) The annual trend of the ratio of the radiographic features of OA-like RA to the number of TKA performed in RA patients.

A significant decreasing trend of the number of TKA was observed during the entire study period (regression coefficient =  − 2.17, 95% CI =  − 3.42 to − 0.91, *p* = 0.006) (Fig. 4a). The annual trend of the percentage of OA-like RA showed a statistically significant increasing trend (*p* < 0.001) (Fig. 4b). The percentage of OA-like RA increased from 20.9% (15/72) in 2006 to 67.7% (20/30) in 2020 (Fig. 4b).

### Comparison between the OA-like RA and conventional RA groups

The results of univariate analysis are shown in Table 1. Among 240 patients included in the analysis, 96 (40%) and 144 (60%) patients were classified into the OA-like RA and conventional RA groups, respectively. The data about age, sex, duration of disease, BMI, dosage of PSL, dosage of MTX, use of bDMARDs, and level of CRP were available in all patients. The data collection rate was 95.4% (229/240) for ESR, 62.9% (151/240) for the presence of ACPA’, and 73.3% (176/240) for DAS28-CRP. All continuous variables were normally distributed. The OA-like RA group had a higher BMI than the conventional RA group (23.8 ± 4.1 kg/m^2^ vs. 22.3 ± 3.6 kg/m^2^, *p* = 0.003). In the OA-like RA group, bDMARDs was used more frequently than that in the conventional RA group (31.2% vs. 18.0%, *p* = 0.017). The breakdown of bDMARDs was 48.2% (27/56) for etanercept, 19.6% (11/56) for abatacept, 16.0% (9/56) for infliximab, 10.7% (6/56) for adalimumab, and 5.3% (3/56) for tocilizumab. Both the levels of CRP and ESR were lower in the OA-like RA group than those in the conventional RA group (CRP: 0.9 ± 1.3 mg/dL vs. 1.6 ± 1.8 mg/dL, *p* = 0.007; ESR: 39.0 ± 23.1 mm/h vs. 49.7 ± 29.5 mm/h, *p* = 0.004).

**Table 1:** Comparison of demographics and clinical data between the OA-like RA and conventional RA groups.

Variables	OA-like RA (*n* = 96)	Conventional RA (*n* = 144)	*p* value
Age, years	66.9 ± 9.8	66.4 ± 9.1	0.74
Male/female	11/133	12/84	0.26
Duration of disease, years	18.4 ± 13.6	18.6 ± 10.5	0.90
BMI, kg/m^2^	23.8 ± 4.1	22.3 ± 3.6	0.003*
Dosage of PSL, mg/day	3.3 ± 2.9	3.8 ± 3.0	0.21
Dosage of MTX, mg/week	4.2 ± 4.2	3.5 ± 3.9	0.21
Use of bDMARDs, *n* (%)	30 (31.2%)	26 (18.0%)	0.017*
Level of CRP, mg/dL	0.9 ± 1.3	1.6 ± 1.8	0.007*
ESR, mm/h	39.0 ± 23.1	49.7 ± 29.5	0.004*
Positive ACPA, *n* (%)	63/82 (76.8%)	49/68 (72.0%)	0.5
DAS28-CRP	2.8 ± 0.8	2.9 ± 0.8	0.53
Remission, *n* (%)	25 (26.0%)	17 (11.8%)	0.36
Low, *n* (%)	16 (16.6%)	10 (6.9%)	
Moderate, *n* (%)	41 (42.7%)	48 (42.1%)	
High, *n* (%)	10 (10.4%)	9 (6.2%)	

Continuous variables are presented as mean ± standard deviation. Categorical variables are presented as number (percentage). *The level of statistical significance was set at *p* < 0.05.

Abbreviations: *BMI* body mass index, *PSL* prednisolone, *MTX* methotrexate, *bDMARDs* biological disease modifying anti-rheumatic drugs, *CRP* C-reactive protein, *ESR* sedimentation rate, *ACPA* anti-cyclic citrullinated peptide antibody, *DAS* disease activity score.

### Multivariate logistic regression analysis

The logistic regression model was used to identify the factors influencing the classification into the OA-like RA group. In addition to the factors that showed significant differences between the groups in the univariate analysis, such as BMI, use of bDMARDs, level of CRP, and ESR, age and sex were included into the model for adjustment. To avoid multicollinearity with CRP, ESR was excluded because both of them are biomarkers of inflammation and showed moderate correlation with each other (R = 0.56, p < 0.001). We selected the level of CRP instead of ESR because several previous studies demonstrated high CRP level as one of the predictors for rapid radiographic progression (RRP)^20–22^. Additionally, the data regarding ESR were missing in some patients, contrary to the perfect data collection in CRP. The logistic regression analysis demonstrated that BMI, use of bDMARDs, and level of CRP were independent factors influencing the classification into the OA-like RA group.

(BMI: OR per unit increase, 1.10; 95% CI, 1.02 to 1.18; *p* = 0.006; use of bDMARDs: OR, 1.94; 95% CI, 1.02 to 3.68; *p* = 0.040; CRP: OR per unit increase, 0.80; 95% CI, 0.67 to 0.96; *p* = 0.020) (Table 2).

**Table 2:** Multivariate logistic regression analysis to determine the factors associated with the classification of knee radiographs into the OA-like RA group.

Variables	Odds ratio	95% CI	*p* value
Age	1.00	0.98–1.03	0.53
Female (vs male)	0.58	0.23–1.43	0.23
BMI	1.10	1.02–1.18	0.006*
Use of bDMARDs	1.94	1.02–3.68	0.040*
CRP	0.80	0.67–0.96	0.020*

*The level of statistical significance was set at *p* < 0.05.

Abbreviations: *BMI* body mass index, *bDMARDs* biological disease modifying anti-rheumatic drugs, *CRP* C-reactive protein, *CI* confidence interval.

## Discussion

The current study showed an increasing trend of radiographs with OA-like RA features in RA patients who underwent TKA during 2006 and 2020. The CNN model was used for the classification of radiographs as OA-like RA and RA, which enabled the objective judgment on radiographs. Moreover, we identified the clinical factors associated with the judgment by the CNN model. The current study suggested that rheumatologists should be aware of the possibility of osteoarthritis if the knee pain is resistant in spite of the adequate treatments using anti-rheumatic drugs.

The results of the current study suggested that the characteristics of knee radiographs from RA patients had been changing from 2006 to 2020. There is a paucity of literatures on the trend of radiographic changes in RA patients. A previous retrospective study conducted at a single hospital investigated the radiographic characteristics of RA patients who underwent primary total joint replacement between 2004 and 2017 and reported that the presence of osteophytes did not differ between two different study periods, namely 2004–2010 and 2011–2017^11^. One of the limitations of this previous study was that the studied joints were mixed up with weightbearing joints such as shoulder and elbow and non-weightbearing joints including hip, knee, and ankle. Additionally, the presence of osteophytes was subjectively judged by two surgeons. We believe that the current study objectively demonstrated the changes in radiographic features of RA patients in the recent decades because the radiographs were automatically classified by the CNN model eliminating the subjectivity of human perception, and it focused exclusively on the knee joint.

The accuracy of the CNN model developed in the current study was higher than that of the previously reported CNN models for the diagnosis of RA. In the current study, the model achieved a training accuracy of 98.3% and a validation accuracy of 95.6% with the radiographs of primary OA. The accuracy of the CNN with 28 layers developed from scratch by Üreten et.al for the classification of hand radiographs as RA and normal was 73.3%^23^. The model for scoring of radiographic finger joint destruction in RA, which was developed from scratch by Hirano et al. with a combination of standard machine learning and CNN, reported the accuracy of 49.3–65.4% for detecting joint space narrowing and 70.6–74.1% for detecting erosion^24^. We consider that the high accuracy of our model might be attributed to the simplicity of the classification task. In the current study, all radiographs used for the training and validation of the model were the radiographs of end-stage arthritis. Notably, we developed the model not for diagnostic purpose or not for using in clinical practice but merely for the investigation in the current study.

We observed a significant decreasing trend of the total number of TKA performed in RA patients at three institutions during the study period. Although the current study may not be the representative of Japanese RA patients, this result was consistent with that of a previous study using a nationwide cohort database, NinJa (National Database of Rheumatic Diseases by iR-net in Japan). The study analyzed the trends of orthopedic surgeries in RA patients from 2004 to 2014 and revealed a decrease in the incidence of orthopedic surgeries during the study period; the greatest reduction (60%) was observed in the prevalence of TKA^5^. The first bDMARD introduced into Japan, infliximab, was approved for the treatment of RA in 2003. As of 2021, eight bDMARDs and five tsDMARDs are available in Japan. In parallel with the raising percentage of patients treated with bDMARDs, which reportedly reached 29.5% in 2018, the remission rate based on DAS28 reached 55.9% in 2018^3^. Contrary to the study based on the Japanese database, a study using the Nationwide Inpatient Sample data from the United States showed a significant increase in TKA performed in RA patients from 2002 to 2013 although the ratio of RA to all primary diseases requiring TKA showed a steady trend^25^. Another trend study using the Nationwide Inpatient Sample data from the United States reported that the prevalence of total elbow arthroplasty and total shoulder arthroplasty in RA patients decreased by 50% and 18%, respectively, from 2002 to 2012^26^. These trends imply a larger effect of pharmacological advances on the reduction of surgeries in non-weightbearing joints than that in weightbearing joints such as knee, which are vulnerable to primary OA. The discrepancy in the trend of the prevalence of TKA between Japan and the United States might be partly attributed to the prevalence of obesity, which greatly influences the occurrence of OA. The prevalence of obesity in Japan is lower than that in Western countries, and the numbers of TKA performed in the general population are fourfold bigger in the United States than those in Japan^9,27^.

A low CRP level was found to be a significant factor for the classification into the OA-like RA group in the current study. Our result was consistent with that of a previous study reporting the correlation between the low CRP level in RA patients and the frequent existence of osteophytes or the amount of osteophytes in knee radiographs before TKA^10,12^. A high CRP level is one of the well-proven prognostic factors for RRP, which is generally defined based on the van der Heijde-modified Sharp score composed of erosion and joint space narrowing scores^20–22,28^. A previous study describing the association between the high CRP level and RRP in RA patients also revealed that a high CRP level had a larger effect on erosion score than that on joint space narrowing score^28^. We could not interpret on which radiographic feature the CNN model focused while judging between OA and RA in the current study. However, we assume that the increased radiographic features of OA represented by osteophytes and decreased radiographic features of RA such as erosions contributed to the increased percentage of the radiographs features of OA-like RA in the recent decades when the number of patients having low level of CRP have been increasing due to the advancement in pharmacological therapy.

The univariate analysis showed no significant difference in DAS28-CRP between the OA-like RA and conventional RA groups regardless of a significant difference in the level of CRP between the groups. In addition to the level of CRP, other elements contributing to DAS28-CRP, such as swollen joint count, tender joint count, and visual analog scale of general health, were considered higher in the OA-like RA group compared to those in the conventional RA group or similar in both the groups. A definitive conclusion could not be drawn because the detailed data regarding each component of DAS28-CRP was not available in all cases. However, all RA patients enrolled in the current study had symptomatic knee arthropathy requiring TKA, and it would be reasonable to assume that both the groups did not have much difference in joint symptoms and visual analogue scale general health score.

In the current study, high BMI was a risk factor for being classified as OA-like RA. This result was consistent with the well-known fact that a high BMI is the risk factor for OA in the general population, particularly for OA in the knee joint^29^. Furthermore, the high BMI at diagnosis in RA patients is associated with an increased lifetime risk of knee replacement^30^. Even in patients with good control of RA disease activity, weight control would be important to escape or delay the need of arthroplasty.

The use of bDMARDs was indicated as an independent factor for the classification as OA-like RA in the current study, suggesting that the blockade of inflammatory cytokines might directly affect the pathology of end-staged arthropathy in RA patients. Bone destruction in joints of RA patients is mediated by the activation of osteoclasts. The inflammatory cytokines targeted by bDMARDs such as TNF-α and IL-6 are known to upregulate the differentiation and bone-resorbing activity of osteoclast. Furthermore, an increased TNF-α level hampers the formation of periarticular osteophyte by DKK-1^31–33^. Several longitudinal studies investigating the effect of TNF-α inhibitors on RA patients have revealed a decrease in bone resorption and an increase in bone formation using bone-turnover makers^34,35^. An increase in the number of RA patients treated with bDMARDs in the recent decades and subsequent alteration of bone-turnover might have contributed to the recent increase in OA-like features in RA patients.

The current study has some limitations. First, although analysis with Grad-CAM revealed that the CNN mainly focused on the joint area, we could not determine in detail which radiographic characteristics the CNN model focused for judging between OA and RA. Second, the clinical data regarding ESR, presence of ACPA, and DAS28-CRP were not available in some cases because the data were collected via retrospective medical chart review. Limited data might have led to undetectable differences in the univariate analysis. Third, we could not deny the possibility that some RA patients might have wrong diagnoses as RA or other comorbid inflammatory arthritis than RA. However, its prevalence was considered not to be biased during the study period. Therefore, we considered its effect on the results of the current study to be little. Fourth, the periods of the radiograph collection for training CNN were different between RA and OA. However, the radiographs in the current study were collected from consecutive patients in the databases and the joint destruction pattern of OA was not thought to have changed during the study period. Therefore, we considered that the difference in data collection period did not bias the results of the current study. In summary, the proportion of RA patients showing OA-like features on knee radiographs who require TKA has been increasing along with the advancement in medical therapy including bDMARDs.

## Data Availability

The datasets generated during and/or analyzed during the current study are available from the corresponding author on reasonable request. The deep learning model used in this study is available in GitHub at https://github.com/takedarort/publish/releases/tag/v1.0.
